# Effects of the COVID-19 pandemic on psychology and disease activity in patients with ankylosing spondylitis and rheumatoid arthritis

**DOI:** 10.3906/sag-2011-188

**Published:** 2021-08-30

**Authors:** Şakir GICA, Yasemin AKKUBAK, Zakire Kübra AKSOY, Adem KÜÇÜK, Erkan CÜRE

**Affiliations:** 1 Department of Psychiatry, Meram Medical Faculty, Necmettin Erbakan University, Konya Turkey; 2 Department of Physiotherapy and Rehabilitation, Faculty of Health Sciences, Necmettin Erbakan University, Konya Turkey; 3 Department of Rheumatology, Meram Medical Faculty, Necmettin Erbakan University, Konya Turkey; 4 Department of Internal Medicine, Ota Jinemed Hospital, İstanbul Turkey

**Keywords:** COVID-19, ankylosing spondylitis, rheumatoid arthritis, psychological well-being, disease activity, coping ability

## Abstract

**Background/aim:**

The COVID-19 outbreak is known to increase stress levels of most patients with chronic diseases. Patients with ankylosing spondylitis (AS) and rheumatoid arthritis (RA) are highly susceptible to environmental stress. In the current study, we aimed to determine how the COVID-19 pandemic psychologically affected patients with chronic progressive diseases such as AS and RA and the effects of these psychological factors on disease activity**.**

**Materials and methods:**

Age and sex-matched patients with AS (n = 80), RA (n = 80), and healthy controls (n = 80) were included in the study. All participants were evaluated with the “Perceived COVID-19 Threat Form (PCTF)”, “Suicide-Ideation Scale (SIS)”, “Hospital Anxiety and Depression Scale (HADS)”, “The Ability to Cope with Trauma (PACT)”, and “Psychological General Well-Being Index (PGWB)” scales. BASDAI was used in patients with AS, and DAS28 was used in patients with RA to assess disease severity.

**Results:**

Compared to healthy individuals, patients with RA and AS had lower PGWB scores and higher HADS depression and anxiety subscale scores. Almost all psychometric assessment test scores were worse in AS patients with high-disease activity compared to those in low-disease activity. PACT scores were higher in patients with moderate RA compared to patients with mild RA (p = 0.006). While a positive correlation was identified between BASDAI and most of the psychometric assessment test scores (r = 0 .36 for PCTF, r = 0.53 for depressive scores, r = 0.54 for anxiety scores, r = 0.57 for suicidal ideation), DAS28 scores were found to be associated only with PACT total and PACT perceived forward-focused subscale scores (r = –.26 and r = .33, respectively).

**Conclusion:**

Psychologically, AS and RA patients were found to be worse off compared to healthy controls. The perceived COVID threat and psychological status were associated with disease activity in AS, but not RA patients. Patients with chronic illnesses may be more vulnerable to the psychological effects of the pandemic, which can worsen disease activity.

## 1. Introduction

Rheumatoid arthritis (RA) and ankylosing spondylitis (AS) are the two most common rheumatological diseases worldwide [1]. It is known that psychiatric symptoms such as depressive mood and anxiety are more widespread in patients with rheumatological diseases than healthy individuals [2–5]. According to data from the United States of America, the lifetime prevalence of psychiatric disorders in RA patients was 63.6% [6]. In a long-term comprehensive longitudinal Swedish cohort study, psychiatric symptoms/illnesses were reported to be more common in patients with AS compared to patients with RA [7]. A significant relationship was reported between psychiatric syndromes and disease activity scores such as Bath Ankylosing Spondylitis Disease Activity Index (BASDAI), Bath Ankylosing Spondylitis Functional Index (BASFI), pain and fatigue in patients with AS [8]. Complaints of anxiety and depression are frequently encountered in these patients [9]. A significant relationship between the scale Disease Activity Score in 28 Joints (DAS28), as well as other disease activity scores and psychiatric syndromes such as anxiety and depression have also been reported in RA [10]. 

The novel coronavirus 2019 (COVID-19) outbreak, which started in Wuhan in January 2019 and then spread worldwide, has been declared a pandemic and its repercussions are continuing. Data from the COVID-19 outbreak as well as previous pandemics have shown that large-scale outbreaks have a wide spectrum of psychosocial effects. In particular, patients with chronic diseases are more susceptible to psychosocial stressors, both physically and psychologically. Symptoms of psychosocial effects such as intense stress, nervousness, anxiety, fear, complaints of depression, decreased tolerance, anger, post-traumatic stress disorder, and psychosomatic complaints are often observed [11,12,13]. Additionally, individuals may obtain incorrect information related to chronic diseases during the pandemic, both through social media and their relatives and friends. This may cause an increase in anxiety levels and a decrease in coping strategies to reduce psychological well-being overall [14]. 

Complaints such as depression, sleep disorders, loss of appetite, and fatigue, which are difficult to adapt to and make it harder to maintain social communication, can be seen more frequently in patients with chronic diseases [15]. Interestingly, the risk of COVID-19 was reported to not be higher in RA and AS patients with no effect on the clinical course of these diseases during the pandemic [16,17]. However, patients with rheumatological diseases, who are prone to psychological disorders, are likely to be easily psychologically affected by the COVID-19 outbreak. Recent studies have shown that patients with RA, axial spondyloarthritis, lupus, and Behçet Disease were severely affected by the COVID-19 pandemic [18–21].

In the current study, we aimed to determine how the COVID-19 pandemic psychologically affected patients with chronic progressive diseases, such as AS and RA and the effects of these psychological factors on disease activity.

## 2. Materials and methods

### 2.1. Participants

Age and gender-matched patients with AS (n=80), RA (n=80), and healthy volunteers (n=80) were recruited for the study. The AS and RA groups were comprised of patients who were followed up at the Necmettin Erbakan University, Meram Medical Faculty, Department of Rheumatology. Patients with AS were contacted by telephone first and related documents were sent to their smart-phones via online communication applications. We excluded patients with other co-morbid chronic diseases from the current study. Similarly, the healthy control group was reached via online communication applications. Relatives of patients with no chronic disease who applied to the internal medicine outpatient clinic were included in the healthy control group. Data from RA patients were obtained with face-to-face interviews at the hospital since blood analysis results were necessary to calculate DAS28 scores. Participants who were under 18 years old, over 70 years old, and with less than 5 years of formal education were not included in the study. Additionally, the data of participants who did not complete all psychometric assessment scales were excluded from the analysis. The sample size was calculated by evaluating the effect size as 0.25, α-err as 0.05, and power as 0.90 with G Power 3.1.9.2 [22,23]. 

### 2.2. Ethical approval 

The Turkish Ministry of Health, General Directorate of Health Services approved the study protocol (Approval Date/Number: 29.04.2020/ŞAKİR GICA-2020-04-29-T16_26_23). The local Ethics Committee on human research also approved the study (IRB Date/Number: 08.05.2020/2020-2485). Prior to the start of the study, all participants received an informed consent form stating the details of the research, and participants who consented to volunteer approved this form. The participants were accepted to the study after they provided their written informed consent.

### 2.3. Data collection tools

Personal Information Form was used to obtain demographic information about the participants. 

Perceived COVID-19 Threat Form (PCTF) was developed by Kavaklı et al [17]. The form has seven items and a five-point Likert type scale ranging from never (score of 1) to always (score of 5). The developers of this scale stated that the form has a one-factor structure according to parallel analysis. The form aims to measure the participants’ perceived COVID-19 threat levels; a higher total score corresponds to a higher perceived threat from the COVID-19 pandemic. Kavaklı et al. reported the Omega reliability score of the scale as .78 [24].

Suicide Ideation Scale (SIS) is a self-reported test that was developed in 1989 by Levine et al. The SIS is comprised of 17 questions that are answered in a yes/no manner, and the total score of the scale varies between 0 and 17. A high score means pronounced suicidal ideation. Nesrin et al. performed the validity and reliability studies for the Turkish scale [25].

Hospital Anxiety and Depression Scale (HADS) was developed by Zigmond and Sinaith to determine risk groups by evaluating anxiety and depression in a brief time in people with physical illness. It is a self-reported scale comprising 17 questions and 2 sub-dimensions. Its validity and reliability in the Turkish have been conducted by Aydemir at al [26].

The Perceived Ability to Cope with Trauma scale (PACT) is a 5-point Likert-type self-reported scale developed by Bonanno et al. (2011) to test the perception of coping with trauma in life [27]. The Turkish form of the scale is comprised of 20 items and has a 2-factor structure. There are no inverse items on the scale. Cronbach’s alpha internal consistency coefficient was calculated as 0.79 for trauma focus, 0.90 for future focus, and 0.79 for the total scale. Ari et al. reported the validity and reliability of the scale in Turkish [28].

Psychological general well-being index score (PGWB) score is a self-reported test that was developed by Diener et al. The score is comprised of eight items that define important elements of human function from positive relationships to feelings of efficacy and a meaningful life [29]. The scores range from 8 (I do not agree with all items absolutely) to 56 (I agree with all items absolutely). A top score indicates the presence of psychological resources and powers in the patient. The adaptation of the scale into Turkish was carried out by Telef et al. [30].

Bath Ankylosing Spondylitis Disease Activity Index (BASDAI) is used in the clinical evaluation of AS patients and is comprised of 6 questions. The patient is asked to answer the questions by considering events during the past week [31] and scores the first five questions between 0 and 10 with 0 corresponding to “absent” and 10 corresponding to “very severe”. The BASDAI score is calculated by summing the average of the scores obtained from the fifth and sixth questions and the scores obtained from the first four questions and dividing the latter score by five. Akkoc et al. reported the validity and reliability study of the Turkish version of the scale [32].

Disease Activity Score-28 (DAS28) is used to evaluate the severity of RA by calculating the swollen, sensitive joint number, “Erythrocyte Sedimentation Rate (ESR)” and “Visual Analogue Scale (VAS)” data together. In some studies, “C-Reactive Protein (CRP)” is also used instead of ESR [33].

### 2.4. Procedure 

After obtaining ethical approval, the data from the study participants were gathered online to keep social distance during the COVID-19 pandemic. All participants received an informed consent form stating the details about the study and individuals who volunteered to take part approved this form. Then, a questionnaire booklet was created with considerations of the order effect. The data of patients with RA were obtained with face-to-face interviews since data from blood analysis were needed to calculate the DAS28 score.

### 2.5. Statistical analysis

Descriptive variables are reported as mean ± SD, median (range), n, and percentage. To evaluate whether the variables had a normal distribution, visual (histogram and probability graphs) and analytical (Kolmogorov–Smirnov and Shapiro–Wilk) tests were conducted. The Kruskal–Wallis test was used for comparison of the suicidal ideation scale scores between the multiple independent groups. The level of statistical significance in nonparametric multiple comparisons was determined with the Bonferroni correction (p < 0.05 / number of comparisons). One-way ANOVA was used for statistical evaluation of the normally distributed numerical data between multiple independent groups. For one-way ANOVA, homogeneity was determined with Levene’s statistic test, followed by a post hoc least significant difference (LSD) test. The RA patients were divided into two groups: a remission or mildly active group (DAS28 < 3.2) and a moderate to high activity group (DAS28 ≥ 3.2) [34]. The AS patients were grouped into high disease activity score (BASDAI ≥ 4) and low-disease activity score (BASDAI < 4) according to the BASDAI scores [35]. Based on the parametric distribution of the data, psychometric assessment scales of remission/mild and moderate RA patients were compared with Student’s t-test and AS patients at remission or non-remission were compared with Mann–Whitney U test. Chi-square test and Fisher’s Exact test were used to compare categorical variables between the groups. If both variables fit the normal distribution, the relationship between the numerical variables was evaluated using Pearson correlation analysis, and if at least one of the variables did not fit the normal distribution, the variables were evaluated using Spearman correlation analysis. For statistical significance, a total type-1 error level of 5% was used.

## 3. Results

Comparative data on sociodemographic characteristics and clinical features of patients with AS, RA, and the healthy control group is shown in Table 1. The mean BASDAI score of patients with AS was found to be 5.3 ± 2.6, and the mean DAS28 score of RA patients was found to be 3.0 ± 0.9. The age and sex of the patient with AS, RA, and control groups were statistically similar. The disease duration in AS patients was 9.0 ± 7.0 years and the same in RA patients was 7.8 ± 6.9 years (p = 0.567). No significant difference was found between the groups in terms of the presence of psychiatric disorders, smoking, pain level, and regular exercise. The level of education was found to be lower in the RA patients compared to AS patients and the control group (p < 0.001). A significant difference in working status was found between the groups (p = 0.001). 

**Table 1:** Comparison of sociodemographic characteristics and clinical features of patients with AS, RA, and healthy control group.

		Patients with AS(n = 80)	Patients with RA(n = 80)	Control (n = 80)	p value
Age (years) (mean ± SD)		41.5 ± 8.8	44.4 ± 9.0	42.4 ± 10.3	0.140
Gender (M) (n, %)		42 (52.5%)	24 (30%)	33 (41.25%)	0.149
Disease duration (years) (median (min-max))		7.0(1–30)	5(0–37)		0.158
BASDAI score (median (min–max))		5.40 (0–10)			
DAS28 score			3.0 ± 0.9		
Pain levels (mean ± SD)		5.4 ± .2.9	5.6 ± 2.8		0.613
Smoking (n, %)		29 (36.25%)	18 (22.5%)	17 (21.25%)	0.858
Education (n, %) a	High school and below	49 (61.2%)	70 (89.7%)	0 (0%)	<0.001 *
	College	31 (38.8%)	8 (10.3%)	80 (100%)	
Working status (n, %) b	Unemployed	29 (36.25%)	47 (58.75%)	3 (3.75%)	0.001 *
	Employed	37 (46.25%)	24 (30%)	61 (76.25%)	
	Temporary incapacity	4 (5%)	3 (3.75%)	9 (11.25%)	
	Retired	10 (12.5%)	6 (7.5%)	7 (8.75%)	
Marital Status (n, %)	Married	70 (87.5%)	66 (82.5%)	68 (85%)	0.676
Individuals who exercise regularly (n, %)		14 (17.5%)	24 (30%)	18 (22.5%)	0.464
Individuals inmate with people at risk (n, %)c		47 (58.75%)	25 (31.25%)	45 (56.25%)	0.003 *
Individuals applying to psychiatry at any time in their life (n, %)		34 (42.5%)	21 (26.25%)	22 (27.5%)	0.909
Individuals with psychiatric illness (n, %)	None	48 (60%)	60(75%)	60 (75%)	0.975
	Depression	5 (6.25%)	6 (7.5%)	5 (6.25%)	
	Anxiety	13(16.25%)	5 (6.25%)	8 (10%)	
	After any trauma	4(5%)	2 (2.5%)	4 (5%)	
	Obsessive–compulsive disorder	1 (1.25%)	0 (0%)	0 (0%)	
	Miscellaneous	9 (11.25%)	5 (6.25%)	3 (3.75%)	

* p < 0.05, One way ANOVA, Chi-Square and Mann–Whitney U tests were performed. AS: Ankylosing Spondylitis, RA: Rheumatoid Arthritis. M: Male, BASDAI: Bath Ankylosing Spondylitis Disease Activity Index, DAS28: Disease Activity Score-28.a In post hoc analysis of education level between 3 independent group p < 0.001 for each analysis. b p < 0.001, when healthy and patients with Ankylosing Spondylitis group were compared; p<0.001, when healthy and patients with Rheumatoid Arthritis group were compared; p=0.020, when patients with Ankylosing Spondylitis and Rheumatoid Arthritis group were compared.c p = 0.749, when healthy and patients with Ankylosing Spondylitis group were compared; p = 0.002, when healthy and patients with Rheumatoid Arthritis group were compared; p = 0.001, when patients with Ankylosing Spondylitis and Rheumatoid Arthritis group were compared.

A comparison of psychometric assessment scale scores of patients with AS, RA and the healthy control group is shown in Table 2. Compared to the control group, patients with RA or AS had lower PGWB scores, and higher HADS depression and anxiety subscale scores (p = 0.024 and p = 0.044 for RA patients; p = 0.001 and p = 0.044 for AS patients, respectively). There was no significant difference in PCTF, SIS, PACT trauma-focused coping scores between the groups. PACT perceived forward-focused subscale scores were significantly lower in RA patients compared to the control group and AS patients. 

**Table 2:** Comparison of psychometric assessment scale results of the patients with AS and RA, and control groups.

	Control(n=80)	Patients with AS (n=80)	Patients with RA (n=80)	p1	p2	p3	p4
PCTF(mean ± SD)	19.4 ± 5.6	20.5 ± 7.1	19.2 ± 6.5	0.429	0.284	0.908	0.242
PGWB(mean ± SD)	45.7 ± 6.9	42.6 ± 10.0	42.1 ± 10.1	0.024 *	0.024 *	0.014 *	0.819
SIS(median [min-max])	1 (0–11)	2 (0–14)	2 (0–15)	0.215	0.141	0.118	0.881
PACT(mean ± SD)	100.7 ± 14.4	96.5 ± 16.9	92.5 ± 17.5	0.008 *	0.105	0.002 *	0.131
PACT trauma-focused coping(mean ± SD)	63.8 ± 13.1	60.7 ± 14.5	59.7 ± 14.7	0.160	0.168	0.067	0.636
PACT perceived forward-focused(mean ± SD)	36.8 ± 7.1	35.7 ± 8.9	32.9 ± 10.5	0.020 *	0.439	0.007 *	0.050 *
HADS depression (mean ± SD)	5.0 ± 3.7	7.1 ± 4.7	7.2 ± 4	0.001 *	0.002 *	0.001 *	0.873
HADS Anxiety (mean ± SD)	6.1 ± 3.7	7.5 ± 4.4	7.8 ± 5.2	0.044 *	0.019 *	0.054	0.679

p1 p value of One way ANOVA or Kruskal–Wallis Test.p2 Healthy controls vs. patients with Ankylosing Spondylitis.p3 Healthy controls vs. patients with Rheumatoid Arthritis.p4 patients with Ankylosing Spondylitis vs. patients with

All psychometric assessment scales were found to be significantly different between AS patients with high-disease activity score and low-disease activity score except the PACT forward-focused sub-dimension. A significant difference was identified only in the PACT total and PACT forward-focused sub-dimension scores between RA patients with mildly active or in remission versus RA patients with moderate to high active. The comparative psychometric assessment scale scores of AS and RA patients according to disease severity are shown in Table 3. 

**Table 3:** Comparison of psychometric assessment scores of patients with AS and RA according to disease activity scores.

	Ankylosing Spondylitis			Rheumatoid arthritis		
	Low-ASDASGroup (n=25)	High-ASDAS Group (n=55)	p	Remitted or Mildly Active Group (n=45)	Moderate or High Active Group (n=34)	p
PCTF	17 (7–35)	21 (7–35)	0.017 *	18.6 ± 6.9	20.2 ± 6.08	0.300
PGWB	48 (8–56)	43 (17–56)	0.031 *	42.8 ± 9.8	41.1 ± 10.5	0.470
SIS	1 (0–6)	2 (0–14)	0.007 *	2 (0–12)	2 (0–15)	0.385
PACT	104 (5–133)	96 (48–140)	0.030 *	87.9 ± 18.1	98.8 ± 14.8	0.006 *
PACT trauma-focused coping	71 (30–84)	59 (24–84)	0.005 *	57.2 ± 16.7	63.0 ± 11.0	0.091
PACT perceived forward-focused	34 (11–49)	36 (16–56)	0.259	30.7 ± 10.2	35.8 ± 10.3	0.035 *
HADS depression	3 (0–17)	8 (1–18)	<0.001 *	6.6 ± 4.0	8.05 ± 3.9	0.135
HADS Anxiety	4 (0–17)	8 (1–21)	<0.001 *	6.8 ± 4.4	8.4 ± 4.3	0.119

* p<0.05, Student T and Mann–Whitney U tests were performed. Abbreviations: ASDAS: Ankylosing Spondylitis Disease Activity Score, PCTF: perceived COVID-19 threat form, PGWB: psychological general well-being index, SIS: suicide ideation scale, PACT: the perceived ability to cope with trauma scale, HADS: hospital anxiety and depression scale.

Correlation analyses indicated the presence of a significant relationship between the BASDAI total score and scores of most of the psychometric assessment scales used. The DAS28 scores were found to be significantly associated only with PACT total and PACT perceived forward-focused subscales scores. Correlation analyses between the BASDAI/DAS28 and psychometric assessment scales are shown in Table 4. Patients with AS showed a negative correlation between PCTF scores and PGWB scores (r = –0.41, p < 0.001) and a positive correlation between PCTF and SIS, HADS-depression subscale, and HADS-anxiety subscale (r = 0.40, p < 0.001; r = 0.36, p = 0.001; r = 0.39, p < 0.001, respectively). However, no correlation was found between PCTF and PGWB scores in patients with RA. Nevertheless, similar to patients with AS, a positive correlation was found between PCTF and SIS, HADS-depression subscale, and HADS-anxiety subscale (r = 0.34, p = 0.003; r = 0.23, p = 0.005; r = 0.48, p < 0.001, respectively) in patients with RA.

**Table 4:** Correlation analysis results between BASDAI / DAS28 scores and psychometric assessment scale scores.

	BASDAI		DAS28	
	r	p	r	p
PCTF	0.36	0.002	0.03	0.773
PGWB	–0.20	0.088	–0.20	0.078
SIS	0.57	<0.001	0.21	0.063
PACT	–0.15	0.362	–0.26	0.026
PACT trauma-focused coping	–0.26	0.027	0.05	0.655
PACT perceived forward-focused	0.14	0.203	0.33	0.003
HADS depression	0.53	<0.001	0.17	0.144
HADS Anxiety	0.54	<0.001	0.19	0.108

Pearson and spearman (rho) correlation tests were performed. Abbreviations: BASDAI: Bath Ankylosing Spondylitis Disease Activity Index, PCTF: perceived COVID-19 threat form, PGWB: psychological general well-being index, COVID-19: novel coronavirus 2019, SIS: suicide ideation scale, PACT: the perceived ability to cope with trauma scale, HADS: hospital anxiety and depression scale.

## 4. Discussion

A pandemic that has spread to large masses worldwide and has caused nearly 2 million deaths will inevitably have a psychological impact on many individuals, including patients with chronic diseases such as RA and AS. In the present study, we investigated the effect of the pandemic on the psychological status and relationship of the latter with disease activities in patients with RA and AS. When compared to healthy controls, we found that patients with RA as well as AS had low levels of well-being and high levels of depression; patients with RA had low ability to cope with trauma, while patients with AS had high anxiety levels. A statistically significant relationship was identified between disease activity and ability to cope with trauma, suicidal ideation, depression, and anxiety levels in patients with AS. AS patients with high disease-activity differ from AS patients with low-disease activity in all psychometric areas. In patients with RA, a significant relationship was identified only between disease activity and the ability to cope with trauma. The ability to cope with trauma of patients with moderate to high active RA was higher than patients with remitted or mildly active RA. We found a positive correlation between perceived COVID-19 threat and suicidal ideations, level of depression, and anxiety in both RA and AS patients. However, a strong correlation was identified between perceived COVID-19 threat and psychological well-being in patients with AS, but not in patients with RA.

Psychiatric disorders such as anxiety, depression, and stress can often be seen in patients with AS and RA [36,37] and can significantly reduce their quality of life by negatively affecting their day to day living [1]. However, psychiatric symptoms may differ in patients suffering from these diseases, and the prevalence of psychiatric symptoms may vary. Sundquist et al. reported that patients with AS had a higher prevalence of psychiatric symptoms compared to patients with RA [7]. In a German study, the frequency of depressive symptoms in patients with axial spondyloarthritis was found to be similar to patients with RA [38]. The HADS anxiety and depression scale scores of both RA and AS patients were reported to be higher than healthy controls [9,39]. The psychological well-being of patients with RA was found to be lower than healthy controls [40]. To our knowledge, ours is the first study evaluating the psychological well-being of AS patients using the PGWB score.

Many patients with rheumatological diseases are likely to be psychologically affected by the ongoing COVID-19 pandemic. Mancuso et al. reported that the psychiatric complaints of patients with rheumatological diseases (a total of 112 patients with Systemic Lupus Erythematosus (SLE), RA, and Spondyloarthritis) increased significantly during the pandemic [18]. On the other hand, Kucuk et al. found that patients with Behçet Disease were psychologically affected by the pandemic, and their PGWB scores were low compared to healthy controls, while the HADS anxiety and depression scores were significantly higher [19]. Tee et al. detected moderate levels of anxiety, depression, and stress in patients with RA during the pandemic [20] but reported that patients with SLE were affected more by the pandemic than patients with RA [20]. Similarly, Picchianti Diamanti et al. found that patients suffering from rheumatological diseases such as RA and AS experienced severe anxiety during the pandemic [21]. Interestingly, Seyahi et al. reported that patients with a rheumatologic disease had lower anxiety and depression levels than hospital workers in a large study involving hospital and academic staff [41]. Previous studies have already shown that healthcare workers experience a high degree of anxiety and fear [42,43]. In contrast to the study by Picchianti Diamanti et al. [21], the current study found that levels of perceived COVID threat between AS and RA patients and healthy controls were similar. These data suggest that the healthy individuals who participated as controls in the study also perceived a similar level of threat of the COVID-19 pandemic as the patients. In our study, the general psychological well-being of patients with AS and RA was worse with higher complaints of anxiety and depression compared to healthy controls. Additionally, a correlation between perceived COVID threat and the suicidal ideation, level of depression, and anxiety were identified in patients with AS and RA suggesting that these patients were severely affected by the COVID-19 pandemic. In fact, the association of a perceived threat with distress and lower levels of well-being has been reported in previous studies conducted on healthy individuals [44]. Similarly, in population studies with large sample sizes, COVID-19 has been reported to increase the frequency of the presence of anxiety and depressive symptoms [45]. 

Whether the follow-up, treatment, and disease activity of rheumatological diseases changed during the pandemic has been the subject of many studies [46,47]. Lopez et al. investigated whether the disease activity scores of patients with RA and axial spondyloarthritis changed during the pandemic period and observed that 37.4% of the patients showed worsening of disease activity [46]. On the other hand, Ciurea et al. found that disease activity of patients with neither RA nor AS increased significantly during the COVID-19 pandemic [47]. The current study did not evaluate whether the disease activity of patients was affected by the pandemic directly. Rather, we found that AS patients with high-disease activity were more affected by the COVID-19 threat compared to patients with low-disease activity; their sense of well-being was lower, and their anxiety and depression scores were higher. At the same time, a significant relationship was found between the perceived threat of COVID-19 and disease activity in patients with AS. Suicidal ideation, ability to cope with trauma, anxiety and depressive symptoms were found to be related to disease activity in patients with AS. However, it was observed that only the ability to cope with trauma was associated with disease activity in patients with RA. There were no patients with severe RA in the current study. The score of coping with trauma was the only difference identified between moderate RA and mild RA cases. The fact that the proportion of AS patients with high-disease activity (68.75%) was higher than the proportion of AS patients with low-disease activity may suggest that patients with AS were more affected by the pandemic compared to patients with RA. Nonetheless, the lack of patients with severe RA may explain the absence of a significant relationship between RA disease activity and psychological parameters. On the other hand, it has been previously reported that psychological symptoms were higher in patients with AS compared to patients with RA [7]. Similarly, in previous studies, it has been reported that disease activity was associated with psychological status in patients with AS [9]. Different clinical courses, age of onset, and gender distributions of the two diseases may result in differences in psychological factors and symptoms. Levels of neurotransmitters such as serotonin and noradrenaline, which play a role in the pathophysiology of depression, anxiety, and pain, may also differ between patients with RA and AS [48–51]. Due to these differences, AS and RA patients may not be affected by the pandemic to the same extent.

An important outcome of the current study is the finding of a more pronounced negative correlation between coping skills and disease activity in patients with RA compared to patients with AS. A significant relationship between the perception of pain and inability to cope has previously been reported in patients with RA [52]. In the same study, it was emphasized that passive coping skills were predictors of both depression and pain in patients with RA, and because RA exacerbations are unpredictable, individuals with inadequate coping skills interpret the disease as uncontrollable [52].

In conclusion, the current study showed that patients with AS and RA displayed a worse psychological status compared to a healthy control group in response to the ongoing COVD-19 pandemic. While the perceived COVID-19 threat was related to most psychometric areas, both psychometric features and perceived COVID-19 threat were related to disease activity in patients with AS. In patients with RA, only the perception of coping with trauma was found to be associated with disease activity. Patients with AS may be more vulnerable to the psychological effects of the pandemic, which can affect disease activity.

## Funding sources

This research did not receive any specific grant from funding agencies in the public, commercial, or not-for-profit sectors.

## Informed consent

Prior to the start of the study, all participants received an informed consent form stating the details of the research, and participants who consented to volunteer approved this form. The Turkish Ministry of Health, General Directorate of Health Services approved the study protocol (Approval Date/Number: 29.04.2020/ŞAKİR GICA-2020-04-29-T16_26_23). The local Ethics Committee on human research also approved the study (IRB Date/Number: 08.05.2020/2020-2485).
